# Performance Investigation of a Flexible Polyvinylidene Fluoride (PVDF) Energy Harvester Array in a Two-Stage Vertical Parallel Configuration

**DOI:** 10.3390/mi17020237

**Published:** 2026-02-11

**Authors:** Yujin Song, Chong Hyun Lee, Jongkil Lee

**Affiliations:** 1 Precision Mechanical Engineering, Graduate School, Gyeongkuk National University, Andong 36729, Republic of Korea; swan1414@naver.com; 2 Ocean System Engineering, Jeju National University, Jeju 63243, Republic of Korea; chonglee@jejunu.ac.kr; 3 Mechanical Engineering Education, Gyeongkuk National University, Andong 36729, Republic of Korea

**Keywords:** PVDF energy harvester, wind speed, vortex flow, uniformity index, vortex amplification index, array efficiency

## Abstract

The performance characteristics of wind-induced energy harvesting were experimentally investigated using a flexible polyvinylidene fluoride (PVDF) energy harvester with a two-stage, parallel, and vertically aligned configuration. Ten PVDF film modules were serially connected to form a single set, and four identical sets were assembled into three different array configurations—2 × 2, 4 × 1, and 1 × 4—to systematically examine the effects of array geometry and vortex interaction on power generation performance. Experiments were conducted at wind speeds ranging from 1 to 3 m/s. At a wind speed of 3 m/s, the 2 × 2 array configuration achieved an average charging voltage of 2.895 V and a total output power of 0.731 W after 600 s, corresponding to approximately 3.3-fold and 4.2-fold increases, respectively, compared with those of the 4 × 1 (0.224 W) and 1 × 4 (0.176 W) configurations. Furthermore, the uniformity index (U = 0.701), vortex amplification index (G = 0.663), and array efficiency (η = 0.789) demonstrate that the 2 × 2 configuration provides the most uniform and efficient energy distribution among the tested configurations. These results indicate that the proposed two-stage parallel funnel-type PVDF energy harvester with a 2 × 2 array configuration is an effective design for high-efficiency energy harvesting, even under low wind speed conditions.

## 1. Introduction

Among various energy harvesting approaches, wind-based energy harvesting utilizes natural airflow as an energy source and offers advantages such as structural simplicity, ease of maintenance, and environmental sustainability. In particular, the development of small-scale energy harvesting technologies optimized for low wind speed environments, where average wind velocities typically range from 1 to 3 m/s, is of critical importance. Accordingly, increasing attention has been directed toward technologies that harvest small amounts of energy from wind-induced vibrations in the surrounding environment and convert them into electrical energy. The development of compact wind energy harvesters capable of operating efficiently under low wind speed conditions therefore has significant practical implications.

Wind-based energy harvesting systems can operate solely on natural airflow without the need for an external power source, and their simple structure and environmentally friendly characteristics have motivated extensive research efforts. Among the various energy conversion mechanisms employed in wind energy harvesting, the piezoelectric approach is one of the most widely adopted. Piezoelectric materials generate electrical output in response to applied mechanical stress or vibration, making them particularly suitable for converting ambient mechanical vibrations into electrical energy in energy harvesting devices [1,2,3].

Polyvinylidene fluoride (PVDF), a polymer-based piezoelectric material, is more flexible and lighter than conventional ceramic-based piezoelectric materials and is therefore well suited for low wind speed energy harvesting applications. Owing to its mechanical flexibility, PVDF can undergo large mechanical displacements even under low-frequency excitation and low wind speed conditions, enabling effective energy conversion in such environments [4,5,6]. Recent studies have focused on enhancing output performance not only through the use of single modules, but also by configuring multiple piezoelectric elements in arrayed structures or by modifying curvature and flow channel geometries to induce vortex shedding and amplify flow-induced vibrations [7].

One of the most representative structural configurations for piezoelectric energy harvesters is the cantilever beam, which is widely employed due to its ability to undergo continuous mechanical deformation. In this configuration, one end of the beam is fixed while the free end vibrates in response to external excitation. When a piezoelectric element is integrated with a cantilever beam, resonance can occur at specific vibration frequencies, leading to significantly enhanced electrical output performance [8,9,10].

PVDF can exhibit larger vibration displacements under identical excitation conditions due to its low elastic modulus, and its ability to achieve stable electrical output in fluid environments such as wind has been experimentally demonstrated. Moreover, PVDF is considered a suitable material for energy harvesters incorporating complex geometries and nonlinear responses, as its mechanical flexibility enables the implementation of various curvatures and expanded array configurations in structural design [8]. Consequently, energy harvesting approaches based on fluid–structure interaction have been actively investigated.

Among these approaches, vortex-induced vibration (VIV) is regarded as a particularly effective excitation mechanism for piezoelectric energy harvesters, as it can generate regular and sustained excitation through periodic vortex shedding formed around bluff bodies such as cylinders or cables [11,12,13]. The capability of vortices to induce continuous vibrations even under low-speed fluid flow in air or water provides a critical technological foundation for the long-term operation of autonomous sensors and unmanned systems.

Previous studies have demonstrated that output performance can be enhanced through geometric optimization of piezoelectric cantilever structures, indicating that structural design plays a crucial role in maximizing the intrinsic performance of piezoelectric materials [9]. For example, significant improvements in output characteristics have been reported for PZT-based cantilever harvesters through shape optimization. However, PZT exhibits inherent brittleness and is susceptible to fatigue under repeated deformation, leading to limited durability. In addition, concerns related to environmental impact and human safety restrict its broader application [14].

In contrast, Lee et al. [7] demonstrated effective power generation under low-speed flow conditions using a PVDF-based energy harvester and further reported that the voltage response of PVDF elements in air could be enhanced by introducing a wind concentration concept [13]. Despite these advantages, PVDF-based harvesters still face challenges associated with relatively low absolute output levels due to the inherently lower piezoelectric coefficient of PVDF compared with ceramic-based materials [4].

To address these limitations, various hybrid and structural enhancement strategies have been proposed. A composite cantilever structure incorporating magnetic coupling was introduced, and a PVDF cantilever-type energy harvester exploiting wake vortices generated behind a cylindrical bluff body was developed [10]. Under experimental conditions of a wind speed of 18 m/s, a magnetic gap of 20 mm, and a mass ratio of 1:2, a maximum output power of 0.095 mW was achieved, and the influence of key structural parameters on output characteristics was quantitatively analyzed.

Another actively investigated approach is the funnel-type energy harvesting (FTEH) structure, which amplifies piezoelectric vibration by accelerating the flow through a converging channel formed by a widened inlet and a narrowed outlet. Lee et al. [7] enhanced the output performance of a PVDF-based FTEH by introducing a spiral flow path, achieving an output power of approximately 39 μW at a flow velocity of 0.25 m/s. Through both numerical simulations and experimental validation, they confirmed that the funnel-type structure operates effectively even under low-flow conditions [7]. Additionally, a funnel-type harvester based on macro fiber composite (MFC) materials was fabricated, and the displacement and output characteristics were analyzed with respect to variations in thickness and length [15]. Notably, the harvested power differed by more than a factor of 20 depending on the stiffness of the support structure and the installation orientation, and an approximately fivefold increase in output was observed when a spiral structure was introduced and vertically mounted on a flexible support. These findings demonstrate that funnel-type structures constitute an important design strategy for overcoming output limitations in low-flow energy harvesting applications.

**Figure 1:**
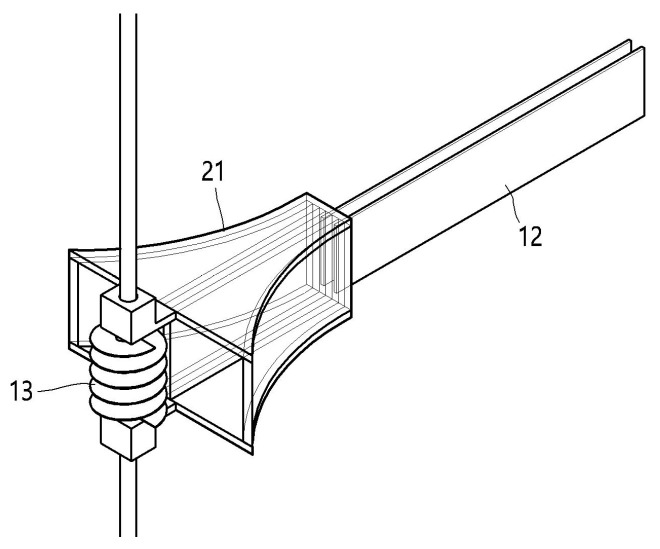
Schematic diagram of the underwater energy harvesting equipment [16].

Furthermore, a United States patented technology entitled “*Funnel-shaped underwater energy harvesting equipment*” (U.S. Patent No. 11637510) proposes a structure in which vortices are generated by a funnel-shaped inlet combined with internal protrusions, and the resulting flow-induced vibrations are transmitted to a piezoelectric element through a collector for energy harvesting [16]. As illustrated in Figure 1, the patented design incorporates multiple piezoelectric elements arranged in the vertical direction, along with a spiral protrusion (spiral screw structure) intended to actively induce vortex formation. This design concept is consistent with recent research on funnel-shaped energy harvesting structures and provides a foundation for further performance enhancement when integrated with array-based configurations.

Another notable research trend focuses on the use of curved cantilever structures. A PVDF cantilever energy harvester with a small radius of curvature was reported to exhibit significantly increased vibration displacement and enhanced output power compared with conventional flat-plate designs [5]. In addition, a cantilever structure incorporating a trapezoidal hollow geometry on a copper substrate was proposed to improve stress distribution relative to traditional flat-plate piezoelectric energy harvesters (PEHs). By mitigating mechanical stress in regions of concentrated deformation, this design simultaneously enhanced output voltage and power density [9]. These studies can be regarded as part of a broader research effort aimed at improving energy harvesting performance through structural shape optimization.

The application of wind concentration structures has also been shown to be effective in low-speed airflow environments. For instance, it was reported that the root-mean-square (RMS) voltage of a PVDF-based harvester could be increased by approximately a factor of two through the use of a flow-concentrating structure [13]. In this approach, the funnel-shaped geometry was designed to concentrate the incoming airflow and increase vibration displacement. Furthermore, a cantilever-type energy harvester combining a vibration–impact mechanism with a triboelectric energy conversion method was proposed, demonstrating stable and efficient energy harvesting performance even under low wind speed conditions [17]. Such multi-modal and hybrid approaches, which integrate complementary structural and transduction mechanisms, are contributing to the expansion of potential applications for wind energy harvesting technologies.

Introducing nonlinear forces or auxetic nonlinear piezoelectric beams can also significantly improve energy harvesting efficiency [18,19]. Research [18] developed a device capable of implementing customized nonlinear forces, demonstrating its feasibility through simulations and experiments. This device allows designers to obtain the most suitable nonlinear force for a given dynamic system [18]. Another paper designed and experimentally verified a nonlinear piezoelectric energy harvester using an auxetic structure, reporting bandwidth expansions of 1556% and 2142% compared to a linear system [19].

Despite these advances, most previous studies have focused on enhancing output performance through single-structure optimization, such as funnel-shaped geometries, curved cantilever designs, or composite structures with cymbal-like configurations. In contrast, this study overcomes the limitations of previous research by installing ten funnel-shaped energy harvesters in series on a pole to maximize their exposure to wind, and by innovatively improving the low-flow response of individual harvesting modules. Furthermore, ten PVDF harvesters were fabricated and electrically connected as a single set, and four such sets (a total of 40 PVDF modules) were arranged in an array configuration to pursue synergistic effects, thus achieving differentiation and originality compared to other studies. This array-based approach allows for performance evaluation beyond single-module studies by utilizing not only the individual funnel-spiral effect of each harvester but also the additional vortex formation and interference effects resulting from the module arrangement. Moreover, the integration with a DPS circuit demonstrates the scalability and practicality of the proposed system, showcasing the originality of this research compared to previous studies that primarily focused on individual harvesting structures.

## 2. Harvester Modeling and Numerical Analysis of Electromechanical Coupled Systems

### 2.1. Electromechanical Coupled System Model

The transverse vibration of a piezoelectric energy harvester with a layered composite cantilever structure, induced by base excitation, is investigated by considering both vibration response and damping effects within the framework of the Euler–Bernoulli beam theory. When a fixed–free cantilever beam is subjected to translational and rotational motion at its base, the transverse displacement at a point x along the neutral axis at time t is denoted as ω(x,t). By accounting for external damping effects, including both viscous and internal damping, the governing equation of motion can be expressed as follows [2]:

(1) $$EI\omega_{,xxxx}+c_{s}I\omega_{,xxxxt}+c_{a}\omega_{,t}+m\omega_{,tt}=0$$

The relative displacement ω(x,t) is expressed as a linear combination of eigenfunctions $\varphi_{n}(x)$ that satisfy the fixed-free boundary conditions [2].

(2) $$\omega \left(x,t\right)=\sum_{n=1}^{\infty }\varphi_{n}\left(x\right)\beta_{n}(t)$$

The eigenmode functions satisfying the fixed-end boundary conditions also satisfy the boundary conditions. Applying the mode coupling equation yields a second-order differential equation for $\beta_{n}(t)$ [2]. Here, $f_{n\;}(t)$ is the external force term for the nth mode, and the total damping ratio, considering both strain rate-based internal damping and viscous air damping, is $\zeta_{n}$. When the base motion is a harmonic function, the steady-state response allows for a quantitative understanding of how the cantilever beam structure responds to base excitation in both the time and frequency domains, which can be used as fundamental data for vibration-based design of piezoelectric energy harvesters. Therefore, the mechanical equation of motion and the circuit equation are as follows [2]:

(3) $${\ddot{\beta }}_{n}\left(t\right)+2\zeta_{n}\omega_{n}{\dot{\beta }}_{n}\left(t\right)+\omega_{n}^{2}\beta_{n}\left(t\right)-{\tilde{\kappa }}_{n}v\left(t\right)=f_{n}(t)$$

(4) $$\dot{v}\left(t\right)+{\left(C_{p}R_{L}\right)}^{-1}+C_{p}^{-1}\sum_{n=1}^{\infty }{\tilde{\kappa }}_{n}\dot{\beta }\left(t\right)=0$$

The two equations above constitute a system of electromechanical equations and are the core model for analyzing the dynamic response of the harvester. Assuming harmonic excitation for time domain analysis, the solution is expressed in the form of complex functions such as $\beta_{n}\left(t\right)=\emptyset_{n}e^{j\omega t}$ and $v\left(t\right)=Ve^{j\omega t}$. By assuming the voltage and displacement responses as complex functions and substituting them into Equations (3) and (4), the following complex-number-based system of equations is derived.

(5) $$\left(\omega_{n}^{2}-\omega^{2}+j2\zeta_{n}\omega_{n}\omega \right)\emptyset_{n}-{\tilde{\kappa }}_{n}V=F_{n}$$

(6) $$\left[{\left(C_{p}R_{L}\right)}^{-1}+j\omega \right]V+j\omega C_{p}^{-1}\sum_{n=1}^{\infty }{\tilde{\kappa }}_{n}\emptyset_{n}=0$$

This system of equations allows for the quantitative analysis of the electromechanical interaction of the piezoelectric harvester and forms the basis for the analysis of output voltage and power. First, when subjected to harmonic excitation, the voltage response is given as follows:

(7) $$v\left(t\right)=\frac{-\sum_{n=1}^{\infty }\frac{{\tilde{\kappa }}_{n}f_{n}}{\left(\omega_{n}^{2}-\omega^{2}+j2\zeta_{n}\omega_{n}\omega \right)}}{\left(C_{p}+\sum_{n=1}^{\infty }\frac{{\tilde{\kappa }}_{n}^{2}}{\left(\omega_{n}^{2}-\omega^{2}+j2\zeta_{n}\omega_{n}\omega \right)}\right)-j{\left(\omega R_{L}\right)}^{-1}}\;e^{j\omega t}$$

Finally, the output power is calculated as the product of voltage and current. This response function can be directly compared with the experimentally measurable output.

### 2.2. Numerical Analysis of Vibration Displacement and Voltage Generation

To investigate the relationship between the vibration displacement of the cantilever beam and the generated voltage in the electromechanically coupled system of a cantilever-based piezoelectric energy harvester, numerical analyses were conducted using Equations (3) and (4), which describe the interaction between the structural vibration and the electrical circuit. In these equations, the damping coefficient and the natural frequency were treated as key parameters influencing voltage generation, and the variations in both the generated voltage and vibration displacement were examined as these parameters were varied.

For the numerical analysis, the equivalent capacitance, load resistance, and electromechanical coupling coefficient were assumed to be constant. Linear models that assume constant electromechanical coupling coefficients and capacitance match experimental results well in the low strain region, but due to the nonlinear coupling characteristics of PVDF, there is a high possibility of significant discrepancies between the model and experiments under high strain (high wind speed) conditions, leading to overestimation of output power, failure to account for resonance frequency shifts, and distortion of power peaks. In addition, the external excitation force was modeled as a constant term to isolate the effects of the damping coefficient and natural frequency on the electromechanical response of the system.

**Figure 2:**
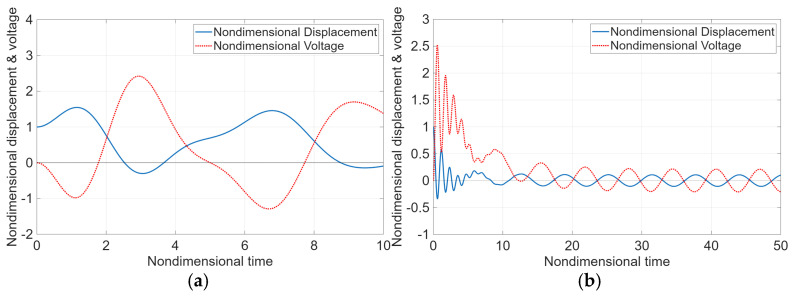
(**a**) Relationship between dimensionless vibration displacement and voltage as a function of dimensionless time when $\;\zeta_{n}$ = 0.1 and $\omega_{n}$ = 1; (**b**) when $\zeta_{n}$ = 0.1 and $\omega_{n}$ = 2.

Figure 2 illustrates the relationship between the dimensionless vibration displacement and the dimensionless voltage as functions of dimensionless time for a damping ratio of $\zeta_{n}=0.2$ and dimensionless natural frequencies $\omega_{n}=1\sim 5$. As shown in the figure, an increase in vibration displacement leads to a corresponding increase in the generated voltage, indicating a strong correlation between mechanical response and electrical output. In Figure 2a,b, although a phase difference between the vibration displacement and the generated voltage is observed, the voltage response remains relatively stable over time. This phase lag reflects the inherent electromechanical coupling behavior of the piezoelectric system and does not significantly degrade the overall voltage generation performance.

**Figure 3:**
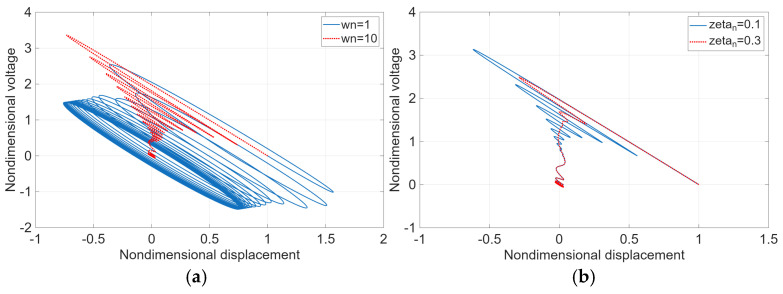
(**a**) Lissajous loop between dimensionless vibration displacement and dimensionless voltage over dimensionless time when $\zeta_{n}$ = 0.05 and $\omega_{n}$ = 1~10; (**b**) when $\zeta_{n}$ = 0.1~0.3 and $\omega_{n}$ = 10.

Figure 3a presents the Lissajous loops between the dimensionless vibration displacement and the dimensionless voltage as functions of dimensionless time for a damping ratio of $\zeta_{n}=0.05$ and dimensionless natural frequencies $\omega_{n}=1\sim 10$. As time progresses, the Lissajous trajectories converge to a stable elliptical loop, as illustrated in Figure 3a, indicating that the electromechanical response of the system reaches a steady-state condition. A comparison between the cases of $\omega_{n}=1$ and $\omega_{n}=10$ reveals that the elliptical loop corresponding to $\omega_{n}=1$ exhibits a significantly larger enclosed area than that of $\omega_{n}=10$. This behavior implies that greater voltage amplitudes are generated at lower vibration frequencies over time. Consequently, to achieve higher voltage output, it is advantageous to exploit the increased voltage generation associated with lower-frequency vibration modes of the energy harvesting device.

Figure 3b illustrates the Lissajous loops between the dimensionless vibration displacement and the dimensionless voltage as functions of dimensionless time for increased damping ratios of $\zeta_{n}$ = 0.1 and $\zeta_{n}$ = 0.3. As shown in Figure 3b, an increase in the damping ratio results in a reduction in the width of the converged Lissajous loop. This trend indicates that higher damping suppresses the vibrational response of the system, thereby leading to reduced voltage generation over time.

**Figure 4:**
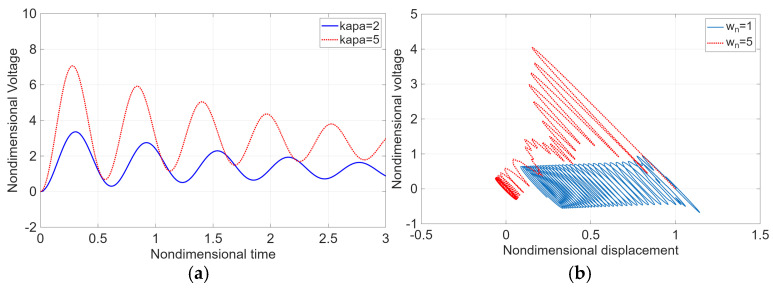
(**a**) Relationship between the increase in the electromechanical coupling coefficient (${\tilde{\kappa }}_{n}=2\;or\;5)$ and the generation of dimensionless voltage when $\zeta_{n}$ = 0.05, $\omega_{n}$ = 10; (**b**) Lissajous loop between dimensionless vibration displacement and dimensionless voltage over dimensionless time when ${\tilde{\kappa }}_{n}$ = 5, $\zeta_{n}$ = 0.05 and $\omega_{n}$ = 1~5.

Figure 4a illustrates the relationship between the electromechanical coupling coefficient, varied from ${\tilde{\kappa }}_{n}=2$ to ${\tilde{\kappa }}_{n}=5$, and the generated voltage under conditions of $\zeta_{n}=0.05$ and $\omega_{n}=10$. As shown in Figure 4a, the generated voltage increases monotonically with increasing electromechanical coupling coefficient ${\tilde{\kappa }}_{n}$, indicating that stronger electromechanical coupling enhances the efficiency of mechanical-to-electrical energy conversion.

Figure 4b presents the Lissajous loops of the dimensionless vibration displacement and the dimensionless voltage as functions of time for a constant damping ratio of $\zeta_{n}=0.05$ and increasing dimensionless natural frequency $\omega_{n}$ from 1 to 5. As $\omega_{n}$ increases, the width of the converged Lissajous loop decreases, as shown in Figure 4b, indicating a reduction in the generated voltage at higher vibration frequencies over time.

The numerical analysis results indicate that selecting a PVDF material with a low damping ratio ($\zeta_{n}$) is essential for enhancing the voltage output of the energy harvester. In addition, reducing the vibration frequency ($\omega_{n}$) from higher to lower values leads to increased voltage generation. A larger electromechanical coupling coefficient (${\tilde{\kappa }}_{n}$) is also shown to significantly improve the generated voltage. Based on these findings, it is anticipated that energy harvesters designed in accordance with these principles can achieve higher conversion efficiency and enhanced overall energy harvesting performance.

## 3. Experimental Setup

### 3.1. Fabrication of an Array-Based Energy Harvester

**Figure 5:**
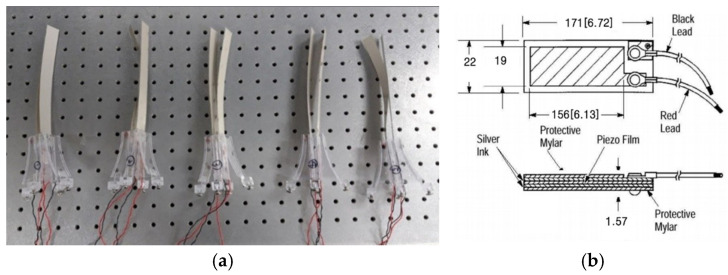
(**a**) A single PVDF harvester module combining a funnel-shaped structure and a spiral screw; (**b**) Configuration of the single PVDF (LDT4-028K/L) and its dimension in mm [inches].

To develop a high-efficiency piezoelectric wind energy harvester capable of effectively generating electrical energy under low wind speed conditions, and to experimentally validate its performance, a vertical array energy harvester employing a two-stage parallel configuration was designed and fabricated using a flexible PVDF, as shown in Figure 5. The funnel-shaped structure and spiral screw were designed based on the conceptual framework of the funnel-shaped underwater energy harvesting equipment reported in Ref. [16], with appropriate modifications to accommodate airflow conditions.

The proposed energy harvester module is based on a conventional funnel-type energy harvester (FTEH) configuration, employing a funnel-shaped geometry with a larger inlet cross-sectional area than that of the outlet. This converging structure exploits the Venturi effect to accelerate the incoming airflow and induce a pressure drop, thereby maximizing the deformation of the PVDF piezoelectric element. In addition, a spiral flow channel was incorporated at the inlet to impart rotational motion to the incoming airflow and promote vortex formation, which further amplifies the vibration displacement of the PVDF.

The structure and specifications of the PVDF film used in this study (Measurement Specialties Inc., Fairfield, NJ, USA, LDT4-028K/L PVDF film) are presented in Figure 5b. The PVDF film has an equivalent capacitance of 11.0 nF and consists of a thin piezoelectric layer sandwiched between polyester protective layers, with silver electrodes deposited on the surface. Electrical signals are extracted through two lead wires, and the film exhibits high sensitivity to bending deformation, enabling efficient conversion of low-wind-speed-induced vibrations into electrical energy.

**Figure 6:**
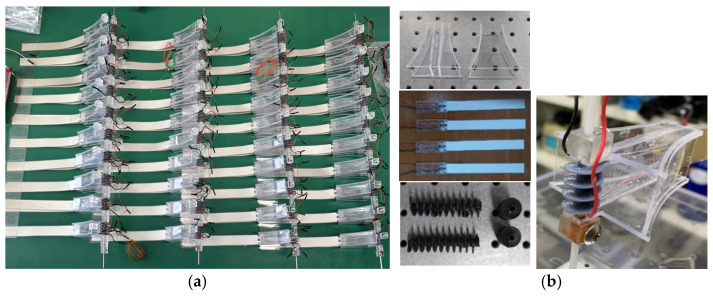
(**a**) Overall view of four sets of PVDF harvester modules, each consisting of 10 modules; (**b**) The photos show the PVDF, funnel shape inlet device, spiral components from top to bottom.

At the outlet of the funnel-shaped inlet, two PVDF films were mounted in a face-to-face configuration to form a single energy harvesting module. Spacers were inserted between the films to prevent mechanical interference during vibration, and the lead wires were arranged to minimize disturbance to the fluid flow. The configuration of a single module, consisting of two opposing PVDF films, is illustrated in Figure 6a,b.

Ten such PVDF harvester modules were vertically arranged at uniform intervals to form a single set. A total of four identical sets were fabricated and assembled into three different array configurations: a 1 × 4 horizontal linear array, a 4 × 1 vertical linear array, and a 2 × 2 grid array. These configurations enabled systematic investigation of fluid flow interference and vortex interaction effects among adjacent modules, as well as a quantitative comparison of output characteristics as a function of array geometry.

### 3.2. Fabrication of the Experimental Setup

**Figure 7:**
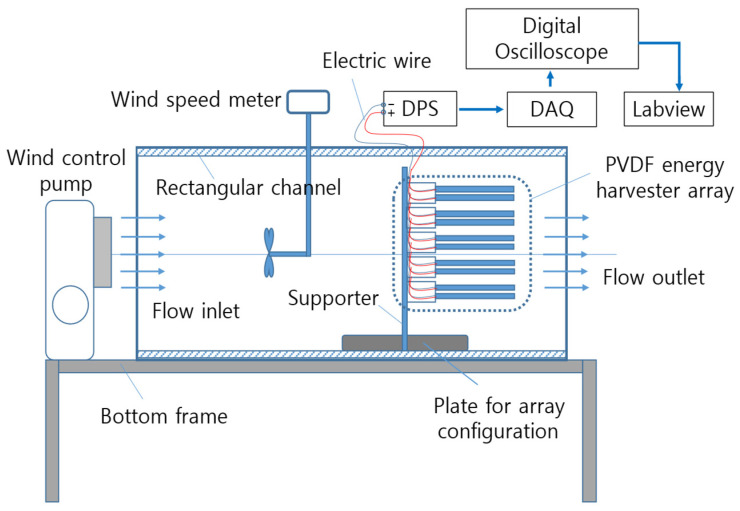
Schematic diagram of the experimental setup.

As shown in Figure 7, the experimental setup consists of a wind control pump, a wind speed meter, a support structure, the harvester array, and a data acquisition system, with the direction of airflow schematically indicated. The blower was adjusted to generate low wind speed conditions in the range of 1~3 m/s.

The support ends of both the anemometer and the harvester module sets were positioned approximately 60 cm downstream from the blower outlet. This configuration enabled accurate calibration of the average wind speed at the inlet of the test section and ensured a relatively uniform incoming flow for all array configurations.

**Figure 8:**
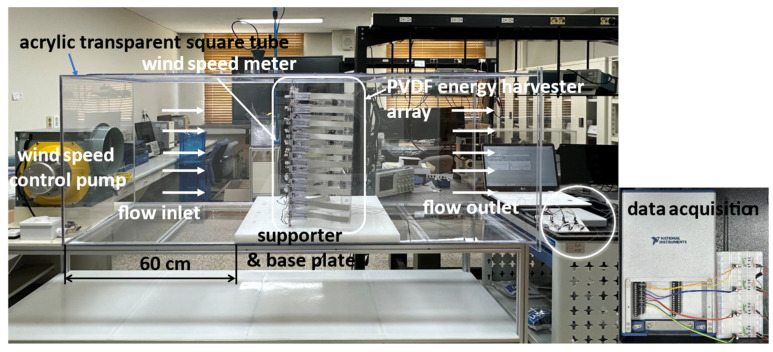
Photograph of the overall experimental setup with DPS (Direct current power supply circuit) circuit and DAQ (National Instruments Co., USB-6351, Austin, TX, USA).

Figure 8 presents a photograph of the constructed experimental setup, illustrating the arrangement of the harvester module sets, the charging circuit, and the data acquisition (DAQ) system, thereby providing a visual overview of the experimental environment. The blower employed in this study is a Sirocco-type ventilation blower (DR-F19DSB) featuring a centrifugal impeller. This configuration generates an asymmetrical jet in the outflow, accompanied by a transverse velocity gradient and shear flow, which simulates the non-uniform flow conditions typically encountered in real-world environments.

The blower conditions employed in this study functionally replicate key characteristics of natural wind. Accordingly, a Sirocco fan-based blowing system was adopted to evaluate the performance of different harvester array configurations under both uniform and realistically non-uniform inflow conditions, ensuring applicability to practical installation environments. The signal acquisition system comprised a DC power supply (DPS) charging circuit and a DAQ system, as illustrated in Figure 8 [20,21]. Data were collected in real time over a duration of 1200 s, with all experiments conducted under identical circuit and load conditions.

**Figure 9:**
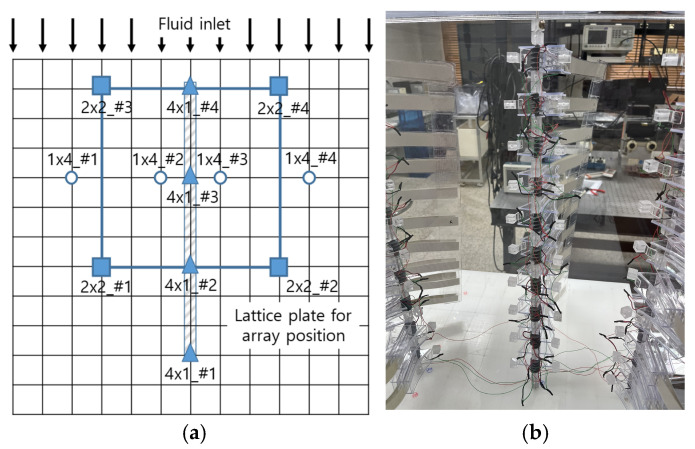
Harvester module support: (**a**) Schematic diagram; (**b**) Photograph of the support.

The harvester module support structure was fabricated from polyoxymethylene (POM), chosen for its excellent durability and dimensional stability. As shown in Figure 9, the support was designed as a modular unit with multiple pre-machined mounting holes for secure module attachment. This design enabled rapid reconfiguration of the array (1 × 4, 4 × 1, 2 × 2) while reliably maintaining module alignment and spacing during repeated experiments. The unit-type support structure minimized deformation induced by vibration, thereby enhancing the reproducibility and reliability of the experimental results.

The described fabrication process and device configuration ensure the experimental validity of this study and provide valuable reference data for the reproducible fabrication and future application of similar structures. In particular, the modular design of the PVDF harvester modules, the flow-amplifying effects of the funnel and spiral screw, and the DPS-based rectification and measurement system collectively establish a standardized experimental platform for research on low-wind-speed energy harvesters [20].

### 3.3. Indicators for Evaluating Energy Harvester Performance

Based on the experimental results obtained at different wind speeds, performance indicators were defined to compare and analyze the charging voltage and output power characteristics of the three array configurations (4 × 1, 1 × 4, and 2 × 2). All experiments were conducted at least four times under identical device and environmental conditions, and the average of the measured data from each trial was used as the representative value.

The charging voltage (V) is defined as the average voltage of the PVDF modules at each position and corresponds to the potential accumulated in the load capacitor (C), as directly measured by the DAQ system. It represents an experimentally recorded quantity rather than a calculated value and provides a quantitative measure of the charge accumulation capacity of the harvester. The output power (P) was subsequently calculated from the measured voltage data under the specified load resistance (R) condition, as defined by the following relation.

(8) $$P=\frac{V^{2}}{R}$$

In this study, considering that the input impedance of the data acquisition system (DAQ) is specified as 50 Ω, a corresponding load resistance of 50 Ω was applied. The high internal impedance of PVDF is a characteristic from a low-frequency and static perspective, while the low resistance value used in this calculation is the result of frequency-based impedance matching under 3 m/s conditions, which is a reasonable choice for obtaining 0.731 W at the maximum power point of the module. Fixed resistance is optimal only at a specific wind speed. In real systems, variable load or a power converter (MPPT) is ideal.

This configuration ensures impedance matching between the harvester output and the measurement system, thereby minimizing signal reflection. All power calculations reported herein were performed under these consistent conditions.

The uniformity index (U) is a performance metric that quantifies the degree of voltage deviation among the four modules in an array. It is defined as the ratio of the standard deviation to the average voltage, as expressed in the following equation:

(9) $$U=1-\frac{\sigma_{V}}{\bar{V}}$$

A U value closer to 1 indicates smaller output variations among the modules, corresponding to a more uniform voltage distribution. Higher U values are generally observed when flow interference or structural asymmetry within the array is minimal.

The Vortex Amplification Index (G) quantifies the degree of vortex-induced voltage enhancement within the array. It is defined as the difference between the maximum and minimum module voltages, normalized by the average voltage, and is calculated using the following expression:

(10) $$G_{vortex}=\frac{V_{max}-V_{min}}{\bar{V}}$$

A higher value of the Vortex Amplification Index (G) indicates a greater variation in flow across different positions within the array, implying that asymmetrical flow patterns—either front-to-rear or left-to-right—are intensified by the array structure. This suggests the presence of strong vortex formation and turbulent interference within the array.

The array efficiency (η) is defined as the ratio of the total power output of the entire array during operation to the sum of the outputs of individual modules, and is expressed by the following equation:

(11) $$\eta =\frac{P_{array}}{\sum P_{single}}$$

A value of array efficiency (η) closer to 1 indicates minimal energy loss during array operation and a structure with reduced inter-array interference. The deviation between repeated experiments was within ±5%, and the average of multiple trials was used to compensate for temporary fluctuations caused by subtle wind speed variations or disturbances. This statistical processing ensured reproducibility and reliability of the measurements, enabling objective comparison of performance among different array configurations.

Experimental data were continuously recorded over 1200 s at a sampling frequency of 100 Hz. Representative values at selected time intervals were extracted to construct the charging voltage curves over time. This approach mitigated the influence of instantaneous vibrations and short-term noise, allowing stable evaluation of the harvester’s long-term charging behavior. Data from 0 to 900 s were used to analyze the convergence pattern, and quantitative comparisons were performed based on the voltage and power at 600 s. This fixed-time evaluation provides a practical metric of energy accumulation before reaching saturation and is particularly relevant for IoT sensors or small wireless devices, where charging time is limited. Previous studies [13] typically used short-term RMS voltage averages (≈5 s) for performance evaluation; the present study extends this to long-term (600 s) charging to assess differences in energy accumulation efficiency among array configurations.

To standardize voltage comparisons across module positions, a consistent numbering system was applied. In the 4 × 1 array, the module closest to the blower outlet was designated #4 and the furthest module as #1. For the 1 × 4 array, modules were numbered sequentially from left to right (#1–#4). In the 2 × 2 array, with the blower facing upward, the numbering was: top left #3, top right #4, bottom left #1, and bottom right #2. This system was applied consistently across all experiments to facilitate analysis of position-dependent voltage characteristics.

Each array was evaluated under two operational conditions: (1) a full-array condition, where all four sets of modules were installed and operated simultaneously, and (2) a single-module condition, where only a specific module was installed. Comparison between these conditions enabled comprehensive analysis of inter-module interference, vortex effects, and the homogeneity of energy distribution within the array.

## 4. Voltage Generation Experiment and Result Analysis

### 4.1. Measurement of Generated Voltage According to Array Arrangement and Wind Speed

#### 4.1.1. Output Characteristics of Different Array Configurations at a Wind Speed of 1 m/s

The charging voltage and output power of the 4 × 1, 1 × 4, and 2 × 2 arrays, each configured in a full-array arrangement, were compared under a wind speed of 1 m/s. At this low wind speed, the incoming airflow momentum is small, limiting the strain amplitude of the piezoelectric films. Consequently, wake interference and recirculating flow induced by the array geometry play a dominant role in determining output uniformity and response dynamics. All experimental results are presented for the time interval from 0 to 900 s, with quantitative comparisons based on the average values measured at 60 s (±1 s).

**Figure 10:**
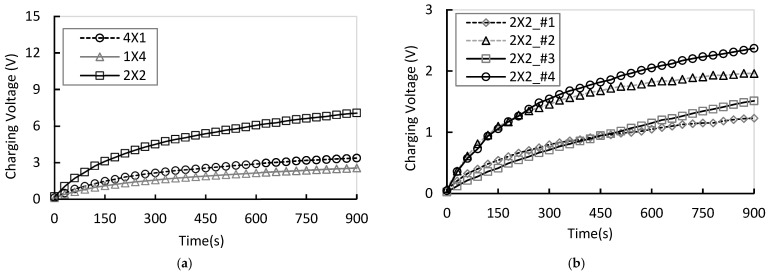
(**a**) Comparison of total charging voltage for each array under 1 m/s condition; (**b**) Comparision of each module charging voltage for 2 × 2 array at 1 m/s.

As shown in Figure 10a, under low wind speed conditions (1 m/s), the 2 × 2 array—where wake energy recirculation effects are dominant—exhibited the highest average voltage (1.517 V) and total power (0.199 W). The uniformity index U was 0.719, indicating the most homogeneous voltage distribution among the modules. In contrast, the 4 × 1 array exhibited significant inter-module voltage variations due to strong axial interference between leading and trailing modules, resulting in a lower uniformity index (U=0.443) and the highest vortex amplification index (G=1.173), indicative of persistent unstable wake flow within the array. For the 1 × 4 array, the average voltage was also low, attributed to increased boundary layer thickness and lateral drift flows near the side walls, which limited overall power generation. Figure 10b presents the voltage generated by each module in the 2 × 2 array at 1 m/s. The maximum voltage was observed at module #4, while the minimum occurred at module #1.

In summary, the 2 × 2 array effectively mitigated wake interference and redistributed the energy more evenly compared to the other configurations, demonstrating superior stability in both spatial uniformity and dynamic response. These results suggest that the two-stage parallel vertical array provides structural advantages by splitting the incoming flow into two rows, spatially attenuating wake effects, and balancing the mechanical strain across all modules.

#### 4.1.2. Output Characteristics of Different Array Configurations at a Wind Speed of 2 m/s

When the wind speed was increased to 2 m/s, the incoming airflow momentum increased by more than fourfold compared to 1 m/s, resulting in a corresponding increase in the strain amplitude of the piezoelectric films. Consequently, both the charging rate and the saturation voltage of each array were generally enhanced. However, the extent of flow recirculation and wake interference remained dependent on the array configuration, leading to notable differences in output uniformity and dynamic response characteristics among the arrays.

**Figure 11:**
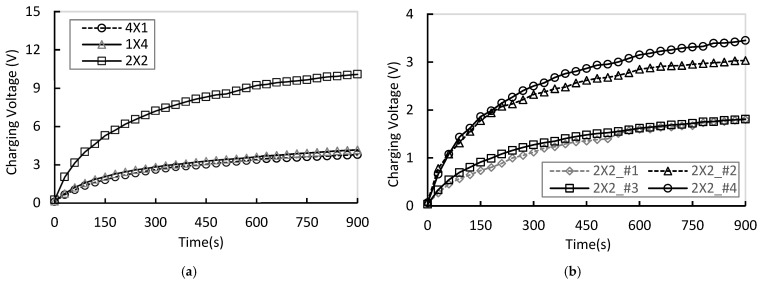
(**a**) Comparison of total charging voltage for each array under 2 m/s condition; (**b**) Comparision of each module charging voltage for 2 × 2 array at 2 m/s.

As shown in Figure 11a, even at a moderate wind speed of 2 m/s, the 2 × 2 array exhibited the highest average voltage (2.305 V) and total power (0.465 W), along with the highest uniformity index (U=0.695) and the lowest vortex amplification index (G=0.672), indicating the most stable voltage distribution among the modules. In contrast, the 4 × 1 array demonstrated reduced output efficiency due to voltage attenuation in downstream modules caused by serial axial interference, while the 1 × 4 array exhibited significant voltage variations between modules, resulting from asymmetric flow induced by lateral drift.

These findings indicate that the two-stage parallel 2 × 2 structure possesses structural advantages that effectively suppress wake interference and maintain stable recirculating flow as wind speed increases, thereby ensuring both high output power and uniform energy distribution. Figure 11b presents the voltage generated by each module in the 2 × 2 array at 2 m/s, with the maximum voltage observed at module #4.

#### 4.1.3. Output Characteristics of Different Array Configurations at a Wind Speed of 3 m/s

When the wind speed was increased to 3 m/s, the kinetic energy of the airflow increased by more than ninefold compared to the 1 m/s condition, resulting in higher fluid loads and mechanical stress on the piezoelectric films. Consequently, the charging rate and saturation voltage of all arrays improved overall. However, significant differences in wake interference and flow recirculation were observed depending on the array configuration. Notably, as 3 m/s represents the maximum wind speed tested in this study, the stability of the vortex structures induced by the array geometry played a critical role in determining both power efficiency and output uniformity.

**Figure 12:**
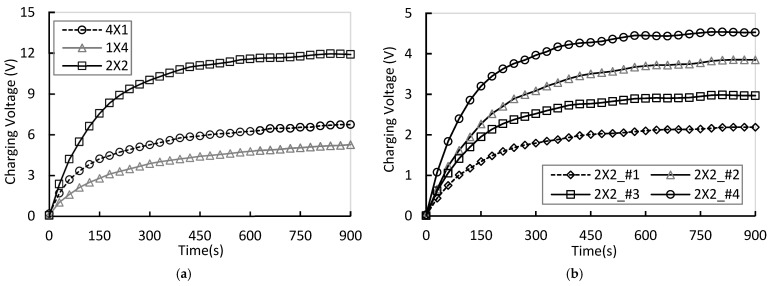
(**a**) Comparison of total charging voltage for each array under 3 m/s condition; (**b**) Comparision of each module charging voltage for 2 × 2 array at 3 m/s.

As shown in Figure 12a, the 2 × 2 array demonstrated the highest performance even under the high wind speed condition of 3 m/s. The average voltage reached 2.895 V, and the total power was 0.731 W, both the highest among all tested arrays. Additionally, the array exhibited the highest uniformity index (U=0.701) and the lowest vortex amplification index (G=0.663), indicating the most stable energy distribution. In contrast, the 4 × 1 array showed reduced power efficiency due to axial interference from leading-edge to trailing-edge modules, whereas the 1 × 4 array experienced significant voltage non-uniformity caused by sidewall boundary layers and lateral drift turbulence.

These results confirm that the two-stage parallel vertical 2 × 2 array is the optimal configuration for this experimental setup, effectively suppressing wake interference and redistributing energy uniformly across all modules. This structure simultaneously enhances both output magnitude and voltage uniformity as wind speed increases. Figure 12b illustrates the measured voltage of each module in the 2 × 2 array at 3 m/s, with the maximum voltage observed at module #4 and the minimum at module #1.

### 4.2. Analysis of Performance Index

Based on the results of the full-scale experiments conducted across the wind speed range of 1–3 m/s, the 2 × 2 array consistently exhibited the highest average voltage, total output power, and superior voltage uniformity among the three tested configurations. Notably, the enhanced performance of the 2 × 2 array was clearly observed even at low wind speeds of 1–2 m/s, experimentally confirming the feasibility of effective energy harvesting under low-speed airflow conditions, which represents a core objective of this study.

**Figure 13:**
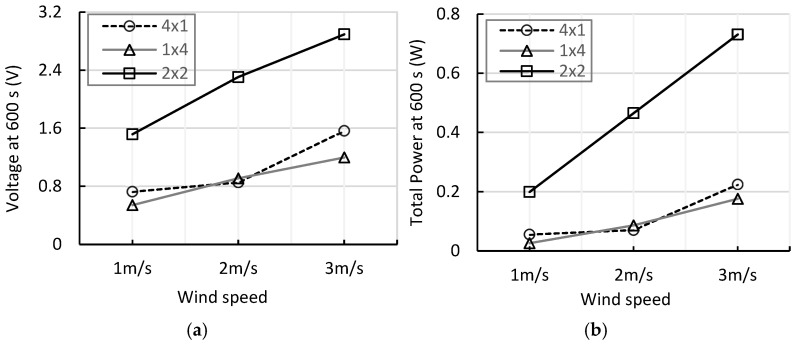
(**a**) Comparison of charging voltage at different wind speeds over 600 s; (**b**) Comparison of total output power for different wind speeds over 600 s.

Figure 13a,b illustrate the average voltage and total output power of each array at 600 s for wind speeds of 1–3 m/s, respectively. The average voltage of the 2 × 2 array increased from 1.517 V at 1 m/s to 2.305 V at 2 m/s and 2.895 V at 3 m/s, as shown in Figure 13a. Correspondingly, the total power exhibited an almost linear increase from 0.199 W to 0.465 W and 0.731 W, as shown in Figure 13b. In contrast, the 4 × 1 array demonstrated more limited increases from 0.725 V → 0.852 V → 1.563 V, while the 1 × 4 array increased from 0.540 V → 0.909 V → 1.197 V.

The superior performance of the 2 × 2 configuration can be attributed to its effective distribution of the incoming flow into two rows, which facilitates wake energy recycling and maintains a balanced angle of attack and vibration phase across all modules. Specifically, at 3 m/s, the average voltage of the 2 × 2 array was approximately 1.9 times higher than that of the 4 × 1 array and approximately 2.4 times higher than that of the 1 × 4 array, while the total power was 3–4 times greater, highlighting the structural advantage of the two-stage parallel vertical arrangement.

Table 1 summarizes the average voltage, standard deviation, uniformity index (U), vortex amplification index (G), and total output power of each array configuration at different wind speeds. The 2 × 2 array consistently maintained the highest voltage and total power across all wind speed conditions, whereas the 4 × 1 and 1 × 4 arrays exhibited comparatively smaller increases in output as the wind speed increased.

These observations can be attributed to a self-balancing mechanism inherent in the 2 × 2 array structure. Specifically, the recirculating vortices formed between the upper and lower modules compensate for reduced strain at the bottom modules and suppress phase differences between the left and right columns. This mechanism not only reduces wake losses but also effectively redistributes energy within the array, minimizing inter-module response disparities. Consequently, power fluctuations are mitigated, and a stable charging behavior is achieved, with saturation voltages converging toward nearly identical values across the modules.

The uniformity index (U) and vortex amplification index (G) exhibited trends consistent with the voltage and power results, further highlighting the advantages of the 2 × 2 array. The 2 × 2 configuration maintained a stable U value of approximately 0.7 across all wind speeds, and the vortex amplification index remained the lowest at 0.65–0.67, indicating stable flow and minimal turbulence interference. In contrast, the 4 × 1 array showed a gradual increase in U from 0.443 to 0.622, but the absolute value remained relatively low, and the vortex amplification index fluctuated from 1.173 to 1.056, suggesting persistent wake-induced instability. The 1 × 4 array displayed a sharp decrease in U from 0.689 to 0.269 and a rapid increase in G from 0.878 to 1.940 with increasing wind speed, reflecting strong asymmetrical flow effects due to lateral drift and boundary layer influence.

**Figure 14:**
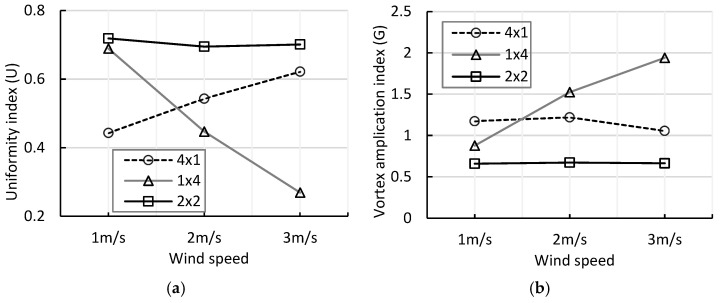
(**a**) Comparison of uniformity index U according to arrangement at different wind speeds; (**b**) Comparison of vortex amplification index G according to arrangement at different wind speeds.

Figure 14a,b show that the 2 × 2 array consistently maintained both uniformity and stability even under low wind speeds of 1–2 m/s, corresponding to typical domestic natural wind conditions with average monthly speeds of 1.5–2.5 m/s. Under these conditions, the 2 × 2 array can achieve sufficient energy density for practical power generation, demonstrating its capability not only in controlled laboratory experiments but also in real-world environments. These results experimentally verify that the two-stage parallel vertical array design provides a stable and efficient energy harvesting platform under varying wind conditions, effectively balancing flow distribution and electrical output across modules.

Furthermore, the 2 × 2 array demonstrated a self-balancing behavior in low-speed wind regions, where the output of the lower modules was compensated through wake recirculation. Even as the wind speed increased, the vibration phases of the modules were naturally maintained, resulting in relatively stable overall energy efficiency. In contrast, the 4 × 1 and 1 × 4 arrays exhibited uneven performance: although the leading or central modules showed increased output with higher wind speeds, the trailing or side-wall-adjacent modules experienced significant output attenuation, limiting the total efficiency improvement. This observation confirms that the two-stage parallel vertical arrangement not only enhances the total harvested power but also stabilizes inter-module interactions, minimizes wake interference, and ensures uniform energy distribution across the array under varying wind conditions.

**Figure 15:**
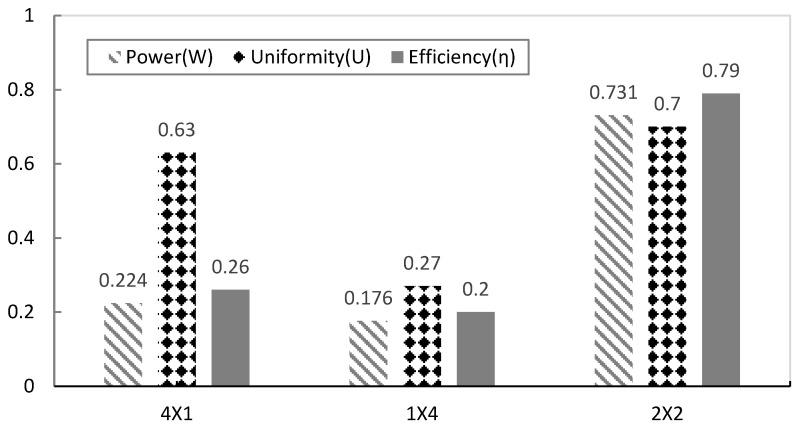
Comparison of key performance indicators for different array structures.

**Figure 16:**
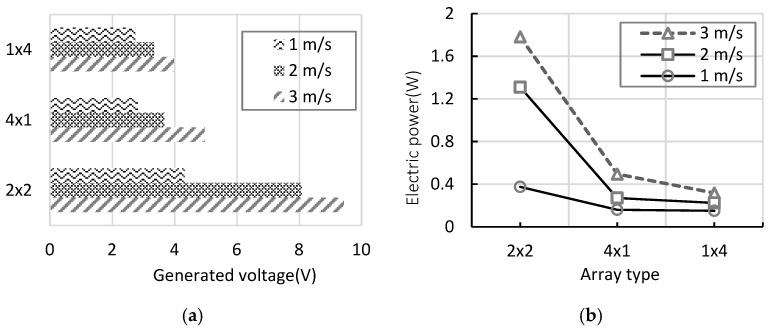
(**a**) Comparison of generated voltage for different array configurations based on wind speed; (**b**) Comparison of generated electric power for different array configurations based on wind speed.

Figure 15 and Figure 16 provide a visual comparison of key performance indicators—output power (W), uniformity index (U), efficiency (η), and voltage—across the three array configurations (4 × 1, 1 × 4, and 2 × 2). The 2 × 2 array consistently exhibited the highest values in all categories, confirming its structural advantages in flow management and energy harvesting. Notably, the efficiency (η) of the 2 × 2 array increased sharply to 0.79, demonstrating that the wake recirculation effect significantly enhanced the total energy recovery. Figure 16 further shows the generated voltage and power across different wind speeds for each array configuration. The 2 × 2 array maintained higher voltage and power outputs than the 4 × 1 and 1 × 4 arrays, highlighting the stability and scalability of this two-stage parallel vertical arrangement under varying low-wind-speed conditions.

In summary, the results demonstrate that the type of flow interference induced by the array configuration critically influences the performance of the energy harvester. The 4 × 1 array, with modules arranged in series along the wind direction, suffered significant wake interference from the leading module, resulting in reduced flow velocity downstream and corresponding power loss. The 1 × 4 array, oriented perpendicular to the wind, avoided serial wake interference but experienced asymmetrical flow due to lateral drift and wall reflections, which weakened the wind in the central region and concentrated energy at the ends. In contrast, the 2 × 2 array exhibited a more complex flow interaction, where upward and downward vortex recirculation, combined with left-right averaging, minimized flow interference while creating a feedback loop that compensated for reduced charge accumulation in the rear modules. Consequently, the 2 × 2 configuration achieved maximized output power and efficiency while maintaining superior uniformity across all modules.

In particular, in the 2 × 2 array, a power reversal phenomenon was observed, where the output of the lower rear modules partially exceeded that of the upper leading modules. This behavior is attributed to local pressure gradient reversal within the wake, where recirculating vortices reduce phase differences across the array, synchronizing the structural response and enabling all modules to reach a stable vibration state. These results indicate that the 2 × 2 array is not merely an expanded-area configuration, but a self-compensating energy harvesting structure that actively corrects flow asymmetry.

Consequently, the 2 × 2 array demonstrated the best performance among the three proposed structures, proving to be the optimal configuration for achieving stable voltage increase and high energy efficiency even in low wind speed environments (1~3 m/s). This provides strong evidence for the effectiveness of a multi-layered, vertically arranged harvester incorporating complex vortex recirculation in the design of future low-speed airflow-based energy harvesting systems.

Overall, the experimental results confirmed that the two-stage parallel vertical PVDF harvester structure effectively recycles wake energy, maintains stable output under low-speed conditions (1~2 m/s), and is less sensitive to flow non-uniformity caused by wind speed fluctuations. These findings support the feasibility of designing piezoelectric harvesters capable of reliable performance in outdoor low-wind environments and highlight the practical potential for powering small self-sufficient IoT sensors or low-power wireless devices.

### 4.3. Discussion

In this paper, under conditions where the wind speed range is not very wide, several factors influence the energy harvester array structure used in the experiment due to the effect of wind passing through the rectangular duct. The limitations of this study include the difficulty in accurately analyzing the experimental uncertainties and errors associated with the array installation due to the influence of turbulence caused by vortex flow between the arrays. However, it is considered satisfactory that the experiments were repeatedly performed for each array to overcome these research limitations and obtain the results.

Considering that the average wind speed range in domestic area throughout the four seasons is 1–3 m/s, higher speeds were not considered due to their impracticality, and the effects of humidity and temperature changes on PVDF performance and vortex formation are considered suitable for more detailed research in the future. Material fatigue, structural durability, performance degradation, and long-term service life are best assessed by deploying the materials in real-world outdoor environmental conditions and collecting data. Since evaluating the durability of PVDF modules requires long-term reliability testing involving repeated loading over extended periods, this paper is limited to verifying initial stability through short-term experiments of 1200 s, and long-term durability evaluation is left as a subject for future research. Furthermore, in repeated experiments, the measured data converged to within 5% of each other, confirming that the data’s significance was not significantly affected. The reason the downstream module performs better than the upstream module is likely because the upstream module generates vortex-induced vibrations, and this directly affects the downstream module. Therefore, it is reasonable to interpret this as an intensification of flow variability and vibrational external forces.

Pressure field and flow visualization can most intuitively demonstrate the synchronization mechanism, and CFD and experimental data (vorticity, velocity field, and periodicity) enable quantitative comparisons. However, the focus of this paper is not on flow visualization but on performance enhancement, so it is not discussed in detail. While the current funnel-and-screw structure is not considered the optimal design, it is a baseline design based on physical principles using previously registered patents, and it allows for focused performance studies on array-level interactions while maintaining the flow guidance and vortex generation mechanisms. The spacing between the arrays determines the degree of vortex interference and wake interaction between the funnel-shaped energy harvester arrays, which in turn affects the power output through flow acceleration and vortex synchronization. The effect of array spacing can be observed through changes in energy harvesting yield as the spacing is adjusted, but in this paper, the spacing was set to a fixed value.

## 5. Conclusions

This study experimentally examined the performance of a PVDF piezoelectric wind energy harvester under low-wind-speed conditions, with particular focus on the influence of different array configurations. Ten single PVDF modules were connected in series to form a set, and these sets were arranged in a two-stage parallel structure (2 × 2) and compared with 4 × 1 and 1 × 4 array configurations. This arrangement allowed for a systematic analysis of the effects of inter-array interference, wake attenuation, and recirculating vortex formation on the power performance of the harvester.

Experiments were conducted at wind speeds of 1–3 m/s, with the charging voltage collected in real time at 100 Hz using a DPS-based charging circuit and DAQ system. Data were averaged over 30 s intervals to minimize noise, and the average of at least four repeated measurements was used as representative data. A constant load resistor and capacitor ensured consistent circuit conditions for fair comparison between arrays.

At a wind speed of 3 m/s, the 4 × 1 array exhibited a typical wake loss phenomenon, where the leading module absorbed most of the flow energy, resulting in a rapid decrease in voltage and power at the rear modules (less than half that of the leading module), with a uniformity index of U=0.622 and array efficiency of 0.26, indicating significant interference losses. The 1 × 4 array, due to its horizontal arrangement perpendicular to the wind direction, suffered from asymmetric flow caused by lateral drift and wall reflections, concentrating energy at the end modules while reducing central module output. Its uniformity was the lowest (U=0.269), with a total output power of 0.176 W.

In contrast, the 2 × 2 array demonstrated superior performance under the same conditions. At 3 m/s, it achieved an average voltage of 2.895 V and total output power of 0.731 W—approximately 3.3 times higher than the 4 × 1 array (0.224 W) and 4.2 times higher than the 1 × 4 array (0.176 W). The array efficiency reached 0.789, with a uniformity index of U=0.701 and vortex amplification index of G=0.663, confirming stable voltage distribution and minimal inter-module deviation.

The experimental results reveal that the superiority of the 2 × 2 array is not merely due to increasing the number of modules in parallel, but rather results from the interaction of upper-lower recirculating vortices and left-right cross-flow vortices. This interaction induces synchronized oscillations across modules, reducing phase differences and enabling reverse charging where downstream vortex energy amplifies the rear modules’ voltage. This self-compensating mechanism actively corrects asymmetries within the array and optimizes the energy flow of the system.

Future research will explore multi-stage grid arrays, such as 3 × 3 and 2 × 3 configurations, extending from the basic 2 × 2 unit. This will enable quantitative analysis of spatial vortex recirculation, pressure field variations, and synchronization conditions, with optimization of funnel channel parameters (curvature, inlet angle, length ratio) to further improve energy harvesting efficiency. Extending the array to a configuration larger than 1 × 4 is expected to amplify energy harvesting due to increased fluid interaction along the sides of the rectangular tubes and enhanced vortex interference effects between the modules.

Overall, the results demonstrate that a two-stage parallel array structure with funnel-shaped and spiral flow channels plays a critical role in the optimal design of low-wind-speed energy harvesters. These design principles provide valuable reference data for the development of small-scale self-powered IoT sensors, low-power wearable devices, and distributed wireless energy systems.

## Figures and Tables

**Table 1:** Performance comparison of array configurations based on wind velocity.

V (m/s)	Array Type	Voltage (V)	Power (W)	U	G
1.0	4 × 1	0.725	0.055	0.443	1.173
	1 × 4	0.540	0.026	0.689	0.878
	2 × 2	1.517	0.199	0.719	0.659
2.0	4 × 1	0.852	0.070	0.543	1.220
	1 × 4	0.909	0.086	0.447	1.524
	2 × 2	2.305	0.465	0.695	0.672
3.0	4 × 1	1.563	0.224	0.622	1.056
	1 × 4	1.197	0.176	0.269	1.940
	2 × 2	2.895	0.731	0.701	0.663

## Data Availability

The original contributions presented in this study are included in the article. Further inquiries can be directed to the corresponding author.

## Nomenclature

$c_{a}$	Air damping coefficient
$c_{s}$	Strain rate damping coefficient
$C_{p}$	Equivalent capacitance
E	Elastic modulus
I	Area moment of inertia
$f_{n}$ **(*t*)**	External force term for the nth mode
$F_{n}$	Magnitude of the nth mode external force term
**FTEH**	Funnel Type Energy Harvester
$G_{vortex}$	Vortex amplification index
j	$\sqrt{-1}$
m	Mass per unit length
**P**	Power
$P_{array}$	Power generated in the array
$P_{single}$	Power generated in a single module
**PVDF**	Polyvinylidene Fluoride
**R**	50 Ω
$R_{L}$	Load resistance
t	Time
U	Uniformity index
V	Voltage amplitude
$V_{max}$	Maximum voltage
$V_{min}$	Minimum voltage
$\bar{V}$	Average voltage
v **(*t*)**	Voltage in a time
***w*(*x*,*t*)**	Relative displacement
$w_{,xxxx}$	$\frac{\partial^{4}w}{\partial x^{4}}$
x	Direction
$\beta_{n}$ **(*t*)**	Vibration coefficient of the nth mode
$\zeta_{n}$	Total damping ratio of the nth mode
η	Array efficiency
$\emptyset_{n}$	Magnitude of the nth vibration coefficient
${\tilde{\kappa }}_{n}$	Electromechanical coupling coefficient
$\sigma_{V}$	Standard deviation
$\varphi_{n}$ **(*x*)**	Mode shape of the nth mode of the beam
ω	Excitation frequency
$\omega_{n}$	Natural frequency of the nth mode
